# Non-Invasive Magnetic resonance imaging Biomarkers to evaLuatE histology-proven kidney fibrosis in Chronic Kidney Disease (NIMBLE-CKD): an observational cohort study protocol

**DOI:** 10.1136/bmjopen-2025-108747

**Published:** 2025-10-29

**Authors:** Dana Kim, Rebecca Kozor, Jason Chen, Muh Geot Wong, Martin Ugander

**Affiliations:** 1 The University of Sydney Faculty of Medicine and Health, Sydney, New South Wales, Australia; 2 Department of Cardiology, Royal North Shore Hospital, St Leonards, New South Wales, Australia; 3 Department of Anatomical Pathology, Royal North Shore Hospital, St Leonards, New South Wales, Australia; 4 Department of Renal Medicine, Concord Repatriation General Hospital, Concord, New South Wales, Australia; 5 Department of Clinical Physiology, Karolinska Institute, Stockholm, Sweden

**Keywords:** Magnetic resonance imaging, Fibrosis, Chronic renal failure

## Abstract

**Abstract:**

**Introduction:**

Chronic kidney disease (CKD) affects 1 in 10 people worldwide and can progress towards kidney failure, which is best predicted by the severity of kidney fibrosis. Currently, kidney fibrosis can only be detected by invasive kidney biopsy which carries procedural risks with limitations on repeat testing. MRI techniques have emerged as potential surrogate markers for kidney fibrosis, though data remain limited. To date, no studies have examined postgadolinium contrast T1 mapping in kidney fibrosis despite its proven utility in assessing myocardial fibrosis. This study aims to develop a multiparametric MRI biomarker including postcontrast imaging to quantify kidney fibrosis in individuals with CKD.

**Methods and analysis:**

In this observational cohort study, a control group of 20 healthy adult volunteers will establish healthy kidney MRI parameters. Two adult non-dialysis CKD cohorts (each n=24) who have undergone kidney biopsy within the last month will derive and validate the MRI models, respectively. Tubulointerstitial fibrosis on kidney biopsy will be assessed by Masson trichrome staining and quantified based on the percentage of cortex affected by blinded pathologists. All participants will undergo a single multiparametric kidney MRI including kidney volumetry, T1 mapping (pre-low-dose and post-low-dose contrast), T2 mapping, T2* mapping, diffusion weighted imaging and phase-contrast MRI of renal artery flow. The primary outcome will be the association between a composite multiparametric MRI marker and tubulointerstitial fibrosis with a minimum variance of 50%. The association between the multiparametric MRI marker and individual MRI variables, and tubulointerstitial fibrosis, estimated glomerular filtration rate and albuminuria will also be studied.

**Ethics and dissemination:**

Ethics approval has been obtained by the Northern Sydney Local Health District Human Research Ethics Committee (2022/ETH00972). Results will be disseminated in relevant peer-reviewed journals and presented at academic conferences.

**Trial registration number:**

ACTRN12622000855729p (Pre-results).

**Strengths and limitations of this study**

- Kidney MRI is a growing area of interest due to its potential to provide a comprehensive assessment of kidney microstructure, haemodynamic properties and function.
- This study combines emerging and novel MRI techniques to develop a non-invasive biomarker for kidney fibrosis which can currently only be quantified histologically by kidney biopsy.
- The multiparametric MRI models used in this study facilitate the examination of multiple MRI techniques including volumetry, T1 mapping, T2 mapping, T2* mapping, diffusion weighted imaging and phase-contrast MRI in a single examination.
- Limitations of this study include its limited sample size and lack of prospective follow-up, thus restricting its ability to report on the prognostic value of the proposed MRI biomarker.

## Background

 Chronic kidney disease (CKD) has a global prevalence of 13%, which is predicted to rise further.^1^ It is an independent risk factor for morbidity and cardiovascular disease and is a leading cause of non-communicable death worldwide.^2^ As such, CKD has recently been recognised as a priority within the WHO non-communicable diseases agenda.^3^ Kidney fibrosis is the strongest predictor and final hallmark of CKD regardless of underlying aetiology.^4^ Early detection and targeted therapy are essential for preventing kidney failure, yet routine clinical markers such as estimated glomerular filtration rate (eGFR) and proteinuria correlate poorly with kidney fibrosis, particularly in early disease.^5^ Currently, the quantification of kidney fibrosis relies on invasive kidney biopsy, which is often performed at advanced stages when irreversible damage has already occurred. Biopsy carries risks with a high incidence of postbiopsy perinephric haematoma and pain,^6^ making repeated procedures unsafe and preventing longitudinal monitoring of fibrosis. Further limitations of kidney biopsy include technical challenges based on patient anatomy and sampling error. Thus, there is a critical need for an alternative non-invasive biomarker to allow for the timely detection and monitoring of kidney fibrosis and identification of individuals at risk of kidney disease progression.

To this end, imaging modalities have emerged as promising tools in the assessment of CKD. In particular, there have been recent advancements in MRI techniques to assess renal function, microstructure, blood flow, perfusion and oxygenation,^7^,^10^ which offer potential for earlier detection of CKD even before clinical manifestations appear. For example, total kidney volume measured from T1-weighted or T2-weighted structural MRI is a validated biomarker in the assessment of kidney function, prognostication and treatment response in autosomal polycystic kidney disease, and has been shown to be associated with kidney function in the general population and other CKD aetiologies.^7 11^ Techniques such as T1 and T2 mapping produce maps over the kidney to quantify tissue-specific T1 and T2 values, respectively, which reflect changes in the molecular environment.^7^ T1 values are sensitive to water content, fibrosis and inflammation, whereas T2 values are more so affected by oedema and inflammation,^6^ and there are a number of ongoing studies to assess their clinical applicability in CKD and kidney transplantation.^12^ T2* mapping uses the paramagnetic properties of deoxygenated haemoglobin to indirectly assess tissue oxygenation^13^ and has been demonstrated to predict the risk of kidney function decline and kidney replacement therapy requirements in CKD.^14^ Diffusion-weighted imaging (DWI) detects the displacement of water molecules in tissue, thus potentially aiding the assessment of fibrosis, perfusion, cellular infiltration and oedema in the kidney.^15^ Phase-contrast (PC) MRI offers a non-invasive method to measure renal blood flow, enabling the assessment of renal haemodynamics in which changes occur at the earliest stages of kidney disease.^16^

A number of these MRI techniques have also been shown to correlate with histologically proven kidney fibrosis, with the most robust data being available for T1 mapping,^17^,^19^ blood oxygen level dependent imaging derived from T2* mapping^20 21^ and DWI.^17^,^2022 23^ However, these findings have been inconsistent with limited sensitivity and specificity and are yet to be validated for clinical application, highlighting the need for further studies and development.^9 24^ Multiparametric renal MRI protocols use multiple MRI techniques from a single examination to provide a comprehensive assessment of renal microstructure and function.^25^ They are proposed to enhance the detection of kidney pathology and have been recommended to be applied in future studies rather than standalone techniques.^7^ Indeed, multiparametric MRI models have been shown to provide greater accuracy in predicting kidney fibrosis compared with single MRI measures.^18^

Importantly, studies of multiparametric renal MRI have avoided the use of gadolinium-based contrast agents (GBCA) to date due to concerns of developing nephrogenic systemic fibrosis (NSF) in CKD. However, a large meta-analysis has demonstrated that newer class II GBCA are safe even in advanced kidney disease,^26^ and recent radiology guidelines no longer necessitate informed consent before administrating class II GBCA regardless of kidney function.^27^ The use of postcontrast MRI has been of particular value in detecting myocardial fibrosis^28^ and is now routinely used in clinical practice. Specifically, areas of myocardial fibrosis are characterised by shorter T1 relaxation times following contrast administration^29^ and greater increases in 1 /T1 (R1) from precontrast to postcontrast, quantified as ∆R1, which is proportional to contrast agent concentration.^30^ Additionally, postcontrast T1 mapping has been shown to correlate with fibrosis on myocardial biopsy,^31^ making this an area with underexplored potential in renal pathology.

Here, we outline an observational study protocol designed to explore several MRI techniques including novel methods using GBCA in multiparametric kidney MRI. The Non-Invasive Magnetic resonance imaging Biomarkers to evaLuatE histology proven kidney fibrosis in Chronic Kidney Disease (NIMBLE-CKD) study aims to develop a non-invasive imaging biomarker for kidney fibrosis in individuals with CKD.

## Methods and analysis

### Study design

**Figure 1:**
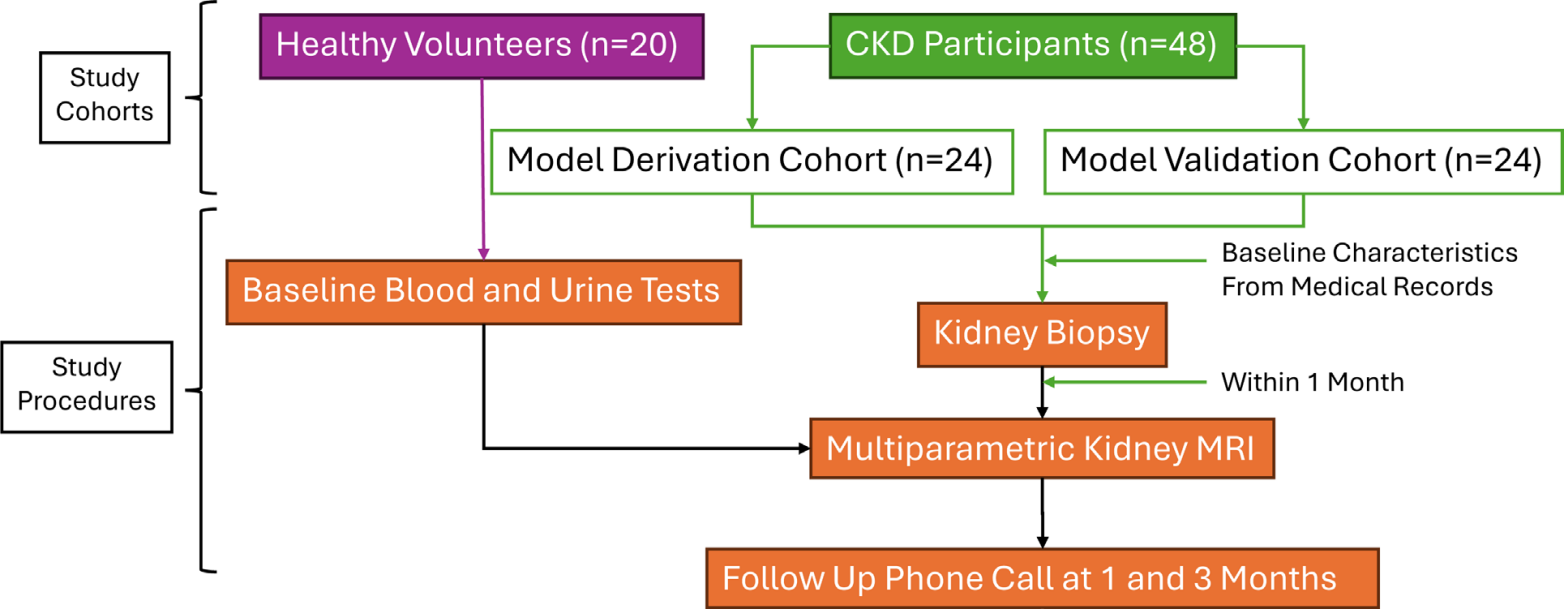
NIMBLE-CKD study cohorts and timeline. CKD, chronic kidney disease; NIMBLE, Non-Invasive Magnetic resonance imaging Biomarkers to evaLuatE histology proven kidney fibrosis.

The NIMBLE-CKD study is an observational cohort study that aims to develop a novel non-invasive MRI biomarker to predict the percentage of kidney fibrosis quantified by histology in individuals with CKD. A control group will consist of healthy volunteers to establish normal kidney MRI parameters. This cohort will not undergo kidney biopsy due to unacceptable risk in a healthy population. An MRI model will be derived from a cohort of individuals with CKD who have undergone kidney biopsy, which will be validated against a second CKD participant cohort. CKD participants will be recruited consecutively and randomly assigned to either the derivation or validation cohorts, respectively. The study cohorts, timeline and procedures are outlined in figure 1.

### Study population

Healthy volunteers will be identified and recruited locally. Potential CKD participants planned for kidney biopsy due to clinical indications will be identified by their treating nephrologists. The inclusion criteria for healthy volunteers include age ≥18 years, no chronic medical conditions, eGFR >90 mL/min/1.73 m^2^ and absence of haematuria or proteinuria on urinalysis. The inclusion criteria for CKD participants include age ≥18 years, eGFR >15 mL/min/1.73 m^2^ and having undergone kidney biopsy within 1 month. Exclusion criteria for all participants include a contraindication to MRI (eg, MRI incompatible implantable devices, claustrophobia), contraindication to GBCA, and pregnant or breastfeeding females. Additional exclusion criteria for CKD participants include dialysis-dependent CKD and rapidly progressive glomerulonephritis on kidney biopsy.

### Data collection

#### Baseline characteristics

Baseline characteristics of all participants including age, height, weight, serum creatinine and eGFR based on the CKDEpidemiology Collaboration (CKD-EPI) formula^32^ will be collected and recorded. Additional clinical characteristics for CKD participants will be obtained from medical records including blood pressure, haemoglobin, urinary albumin excretion (spot albumin to creatinine ratio), presence of haematuria, medical comorbidities and concomitant medications including diuretics, renin–angiotensin system inhibitors and sodium-glucose cotransporter-2 inhibitors. Baseline characteristics for each cohort will be reported and compared.

#### Kidney biopsy and pathology analysis

Percutaneous kidney biopsy will be conducted using ultrasound or computed tomography guidance based on the treating physician’s discretion. Histopathological samples of eligible participants will be reviewed prior to proceeding with MRI to ensure the exclusion criteria are not met.

All histopathological samples will be visually assessed and scored by two independent renal pathologists, who will be blinded to the MRI results. Tubulointerstitial fibrosis (TIF) will be assessed by Masson trichrome staining and quantified based on the percentage of cortex affected. The degree of TIF will be scored as a continuous variable from 0% to 100% and reported as the average of the two values from each pathologist. Based on this score, TIF will also be categorised based on the standard clinical scoring system (TIF 0 (<10%), TIF 1 (10%–25%), TIF 2 (26%–50%) or TIF 3 (>50%)). Other features of chronic kidney damage, including glomerulosclerosis, tubular atrophy and arteriosclerosis, will similarly be graded.^33^ The presence of primary renal (glomerular, tubular, interstitial and vascular) pathology and the formal histopathological diagnosis will also be recorded.

#### MRI acquisition

**Figure 2:**
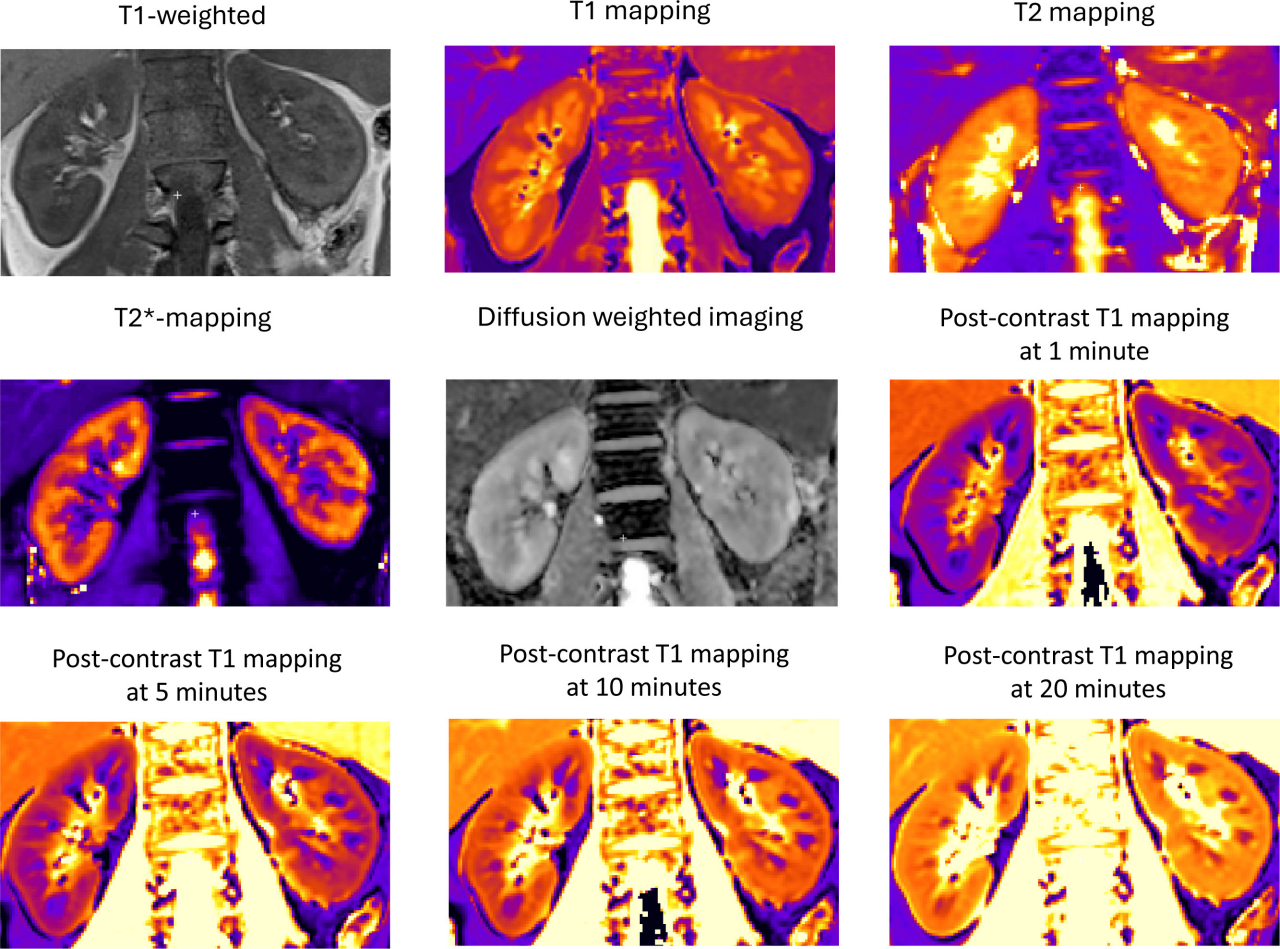
Representative multiparametric MRI acquired for the NIMBLE-CKD study. Example of different MRI techniques in the NIMBLE-CKD study acquired in a chronic kidney disease participant. NIMBLE-CKD, Non-Invasive Magnetic resonance imaging Biomarkers to evaLuatE histology proven kidney fibrosis in Chronic Kidney Disease.

All MRI will be acquired using a 3.0 Tesla MRI scanner with an 18-channel body array coil and an integrated 32-channel spine coil as the receiver (Siemens Healthcare, Erlangen, Germany). Multiparametric renal MRI (figure 2) will be conducted over 45 min. Participants will be fasted for 4 hours and positioned supine with arms placed alongside the body.

Localisers in the axial, coronal and sagittal planes will be obtained for renal imaging. Coronal T1-weighted volumetric interpolated breath-hold examination (VIBE) sequences using a Dixon technique will be acquired to measure kidney volume. The acquisition parameters will include: slice thickness=1.5 mm, repetition time (TR)=4.21 ms, echo times (TE)=1.34 ms (out-of-phase) and 2.57 ms (in-phase), flip angle=9°, field of view (FOV)=260×320 mm, in-plane resolution=1.25×1.25 mm^2^. Coronal T1 mapping will be performed using a modified Look-Locker inversion recovery (MOLLI) sequence with a 5 (3)3 acquisition scheme during breath-hold. The parameters will include slice thickness=8 mm, TR=401.68 ms, TE=1.04 ms, flip angle=35°, FOV=218 × 256 mm, in-plane resolution=1.48×1.48 mm^2^. Coronal T2 maps will be acquired using a T2-prepared steady-state free precession with the following parameters: slice thickness=8 mm, TR=317.95 ms, TE=1.25 ms (T2 Prep Duration 0, 35, 55 ms), flip angle=12°, FOV=154×192 mm, in-plane resolution=1.98×1.98 mm^2^. Coronal T2* maps will be acquired using a multi-echo gradient echo (GRE) sequence which will include the parameters: slice thickness=8 mm, TR=54.95 ms, TE=1.22, 2.36, 3.19, 4.30, 5.13 and 6.24 ms, flip angle=20°, FOV=156×192 mm, in-plane resolution=1.98×1.98 mm^2^. Coronal DWI will be performed using single-shot Echo Planar Imaging with Spectral Attenuated Inversion Recovery fat suppression during free breathing with b-values of 50 and 800 s/mm^2^ and the following parameters: slice thickness=8 mm, TR=3500 ms, TE=68 ms, flip angle=90°, FOV=216×268 mm, in-plane resolution=1.42×1.42 mm^2^. PC imaging will be performed in the through-plane perpendicular to the renal artery at the level of the hilum using a 2D GRE sequence with PC-velocity encoding (VENC) set at 100 cm/s. The imaging parameters will include: slice thickness=6 mm, TR=38.40 ms, TE=2.65 ms, flip angle=20°, FOV=192×265 mm, in-plane resolution=1.41×1.41 mm^2^.

Gadoteric acid, a class II macrocyclic agent, will be administered via a peripheral intravenous cannula at a dose of 0.05 mmol/kg. No unconfounded cases of NSF have been reported with gadoteric acid.^34^ Postcontrast T1 maps will be obtained at 1, 5, 10, 15 and 20 min following contrast infusion using the T1 mapping parameters outlined above. All imaging acquisition parameters are detailed in table 1.

**Table 1:** MRI techniques and acquisition parameters

Imaging acquisition parameter	MRI technique						
	T1-weighted	T1 map	T2 map	T2* map	DWI	Phase contrast	Postcontrast T1 map
Sequence	VIBE	MOLLI 5 (3)3	T2-prepared SSFP	Multiecho GRE	ss-EPI	PC-VENC GRE	MOLLI 5 (3)3
Orientation	Axial and coronal	Coronal	Coronal	Coronal	Coronal	Through-plane perpen-dicular to the renal artery	Coronal
Respiratory control	Breath-hold	Breath-hold	Breath-hold	Breath-hold	Free-breathing	Breath-hold	Breath-hold
Slice thickness (mm)	1.5	8	8	8	8	6	8
Slices (number)	120	3	3	3	10	1	3
TR (ms)	4.21	401.68	317.95	54.95	3500	38.40	481.68
TE (ms)	1.34 (out of phase) 2.57 (in-phase)	1.04	1.25 T2 Prep Duration 0, 35, 55	1.22, 2.36, 3.19, 4.30, 5.13, 6.24	68	2.65	1.04
Flip angle (°)	9	35	12	20	90	20	35
FOV (mm)	260×320	218×256	154×192	156×192	216×268	192×265	218×256
In-plane resolution (mm)	1.25×1.25	1.48×1.48	1.98×1.98	1.98×1.98	1.42×1.42	1.41×1.41	1.48×1.48

DWI, diffusion-weighted imaging; FOV, field of view; GRE, gradient echo; MOLLI, modified Look-Locker inversion recovery; PC-VENC, phase-contrast velocity encoded segmented; ss-EPI, single-shot Echo Planar Imaging; SSFP, Steady-State Free Precession; TE, echo time; TR, repetition time; VIBE, volumetric interpolated breath-hold examination.

#### MRI analysis

MRI will be analysed using the image analysis software Segment (Medviso, Lund, Sweden) by two independent observers blinded to the participant’s baseline laboratory and biopsy results. The first analysis of observer 1 will be used for model derivation. Intra-observer variability will be evaluated and reported following second analysis by observer 1 of 5 healthy volunteers and 15 CKD participants (total n=20) that are randomly selected. Interobserver variability will be evaluated in those same 20 participants comparing observer 1 with observer 2. All analyses will be repeated for the left and right kidneys for each participant. Total kidney, cortical and medullary volume will be measured by manually placing regions of interest (ROIs) on each slice of the coronal T1-weighted VIBE in-phase images. Motion-corrected images will be used to measure T1, T2, T2* and postcontrast T1 values. Apparent diffusion coefficient (ADC) maps will be generated from DWI. A single ROI will be manually drawn on the T1, T2, T2*, postcontrast T1 and ADC maps in the kidney cortex and medulla, avoiding areas of heterogeneity, vascular structures, cysts, haemorrhage and artefacts. Cortical and medullary values will be reported, alongside the cortical-medullary difference and cortical-medullary ratio. Cortical and medullary contrast distribution measures will be quantified from postcontrast T1 mapping images over time. Relative concentrations of the extracellularly distributing and renally excreted GBCA will be quantified in the renal cortex, renal medulla and blood pool over time as ∆R1, where 1 /T1=R1 and ∆R1=the difference between R1 before contrast and R1 at a given time point. These relative concentrations have not previously been quantified in health or disease in this manner, and the patterns of cortical contrast distribution dynamics will be evaluated in an exploratory manner using tissue kinetic modelling approaches for potential inclusion into the multivariable model. Renal artery blood flow analysis will be performed using PC scans with static tissue background correction. A manual ROI will be placed on the renal artery across all phases of the cardiac cycle. Renal perfusion will be calculated by dividing the mean renal artery blood flow by total kidney volume.

### Outcomes

The primary outcome will be the association between a multiparametric MRI marker and TIF quantified on kidney biopsy with a minimum variance of 50% (R^2^=0.5). The model for the primary outcome will include the following MRI variables: total kidney volume, cortical T1 value, cortical T2 value, cortical T2* value, cortical ADC value, renal artery blood flow, renal perfusion and cortical contrast distribution measures.

The secondary outcomes will include the association between (1) individual MRI variables and TIF, (2) combinations of MRI variables and TIF, based on significant correlations identified in (1), (3) the multiparametric MRI marker for the primary outcome and eGFR and (4) the multiparametric MRI marker for the primary outcome and albuminuria. Exploratory outcomes will include the association between (1) the multiparametric MRI marker for the primary outcome and TIF as a categorical variable and (2) individual MRI variables and TIF as a categorical variable.

### Sample size calculation

There will be 20 participants in the healthy volunteer cohort. To detect a minimum of 50% variance (R^2^) explained by eight MRI variables, at a 0.05 level of significance and minimum power of 0.80, a sample of 24 subjects is required. Therefore, there will be 24 participants in each of the CKD participant derivation and validation cohorts.

### Data analysis plan

All statistical analyses will be performed using R Statistical Software (V.4.1.3; R Core Team 2022). Continuous variables will be expressed as mean and SD and compared using t-tests for normally distributed data. Skewed data will be expressed as median and IQR and compared using Mann-Whitney U tests. Data normality will be assessed visually using histograms. Categorical data will be compared using χ^2^ tests. The healthy control group will determine normal MRI reference ranges for baseline comparison. All variables (MRI, TIF, eGFR and albuminuria) will be measured and reported as continuous variables for the primary and secondary outcomes. TIF will be reported as a four-level categorical variable for the exploratory outcomes.

For the primary outcome, a multivariable linear regression model including all MRI variables will be fitted to examine the association between the multiparametric MRI marker and TIF. The model will be tested and developed in the CKD participant derivation cohort. Model performance will be assessed using a prespecified minimum variance (R^2^) threshold of 0.50 and p<0.05. Patterns of missing data will be inspected and reported. Where appropriate, multiple imputation will be used to account for missing data under the missing at random assumption, and sensitivity analyses based on complete cases will be performed. A second independent CKD participant validation cohort will be used to evaluate the accuracy and sensitivity of the model using adjusted R^2^. The multivariable linear regression model will be derived using elastic net regularisation and nested resampling cross-validation in order to minimise overfitting. The agreement performance of the model will be reported using adjusted R^2^ with 95% CIs. Similar univariable and multivariable linear regression models will be fitted to examine the association between the MRI variables and TIF, eGFR and albuminuria for the secondary outcomes. All statistical tests will be two-sided and performed at a 0.05 level of significance.

### Safety monitoring

All serious adverse events that occur during the study will be documented by the Investigator and reported to the relevant governing bodies. Additional reportable GBCA-associated adverse events include serious allergic-like reactions requiring treatment or hospitalisation, and NSF within 3 months of contrast administration.^35^ Complications related to kidney biopsy will not be documented for the purpose of this study as this procedure is within the scope of standard clinical practice and will be managed accordingly by the participant’s treating nephrologist.

### Patient and public involvement

The George Institute for Global Health Consumer Kidney Panel was involved in the review of the study methodology, procedures and patient information consent form.

### Study status

Participant recruitment commenced in July 2024 and is anticipated to be completed by December 2028.

### Ethics and dissemination

This study has been approved by the Northern Sydney Local Health District Human Research Ethics Committee (2022/ETH00972), with governance approval from Royal North Shore Hospital (2022/STE01742) and New South Wales Health Pathology (2023/STE03679) and is registered with the Australian New Zealand Clinical Trials Registry (ACTRN12622000855729p). Results will be disseminated in relevant peer-reviewed journals and presented at academic conferences. All manuscripts will be reviewed and approved by all investigators prior to publication. Authorship will be based on contribution to the manuscript.

## Discussion

Early detection of CKD and identification of those at risk of kidney failure is important to guide prognostication and treatment decisions. Current clinical markers for estimating kidney function such as eGFR are notoriously fraught with limitations,^36^ and the assessment of kidney fibrosis by kidney biopsy comes with risk. Here, we propose to develop a non-invasive imaging biomarker for histology-quantified kidney fibrosis and explore its relationship with eGFR and albuminuria. By using multiparametric models, we aim to build on existing literature for MRI techniques including T1 mapping, T2* mapping and DWI and examine emerging techniques such as PC-MRI. We will also provide novel data on the application of postcontrast T1 mapping in kidney MRI and kidney fibrosis which, to the best of our knowledge, has not been previously investigated. By leveraging established techniques in cardiac MRI, the addition of postcontrast imaging alongside previously studied kidney MRI techniques has the potential to profoundly enrich the assessment of kidney fibrosis in CKD.

Limitations including cost, accessibility and inconsistent or limited data have hindered the clinical application of kidney MRI biomarkers to date.^37^ Global collaborative efforts are underway to standardise preclinical kidney MRI protocols,^8 38^ yet knowledge gaps persist. For example, experts have failed to reach consensus on factors such as the impact of medication and hydration or fasting status on MRI and image analysis techniques.^39 40^ Limitations specific to our study include the limited sample size and the use of a single MRI scanner which may preclude direct application of our MRI protocols to other scanners. As the CKD participants will only include patients who have undergone kidney biopsy, there is potential risk for selection bias where patients with conditions that do not meet biopsy criteria are not represented, thus limiting generalisability. Furthermore, as we will be acquiring a single MRI examination per participant, we will be unable to assess disease progression or report on the prognostic value of these MRI models at this stage.

Despite these limitations, kidney MRI remains an evolving field of great interest due to its ability to provide a wide breadth of information regarding kidney microstructure, haemodynamic properties and function. While multiple imaging techniques have been studied in CKD, MRI has the most data in detecting kidney fibrosis such that it is the only modality to be used as an imaging endpoint in clinical trials.^41^ Certainly, the non-invasive nature of MRI and ability to be safely repeated over time makes it attractive for longitudinal monitoring of disease. In addition, with the focus on anti-fibrotic therapies in the prevention of CKD progression,^42 43^ the conceivable use of MRI to assess efficacy in reducing kidney fibrosis to further drug development is highly anticipated. The NIMBLE-CKD study aims to advance our ability to assess kidney fibrosis and serve as a platform for larger prospective studies to ultimately demonstrate the translational potential of multiparametric kidney MRI biomarkers for kidney failure risk stratification in clinical practice and surrogate kidney outcomes in clinical trials.
