# Supplementary material for: Non-Invasive Magnetic resonance imaging Biomarkers to evaLuatE histology-proven kidney fibrosis in Chronic Kidney Disease (NIMBLE-CKD): an observational cohort study protocol
Source: BMJ Open. 2025 Oct 29;15(10):e108747. doi: 10.1136/bmjopen-2025-108747 (PMC12581042; doi:10.1136/bmjopen-2025-108747)
Supplement: Supplementary file 1 [file bmjopen-2025-108747.reviewer_comments.pdf]

PEER REVIEW HISTORY

BMJ Open publishes all reviews undertaken for accepted manuscripts. Reviewers are asked to complete a checklist review form and are provided with free text boxes to elaborate on their assessment. These free text comments are reproduced below.

ARTICLE DETAILS

Title (Provisional)

Non-Invasive Magnetic resonance imaging Biomarkers to evaLuatE histology-proven kidney fibrosis in Chronic Kidney Disease (NIMBLE-CKD): an observational cohort study protocol

Authors

Kim, Dana; Kozor, Rebecca; Chen, Jason; Wong, Muh Geot; Ugander, Martin

---

VERSION 1 - REVIEW

**Reviewer**	**1**
**Name**	**Torres, Ulysses**
**Affiliation**	**Universidade Federal de São Paulo, Department of Radiology**
**Date**	**08-Sep-2025**
**COI**	**None**

This protocol describes the NIMBLE-CKD study, an observational cohort designed to validate multiparametric MRI biomarkers for assessing histology-proven renal fibrosis in chronic kidney disease (CKD). Three cohorts will be included: healthy volunteers (n=20) to define normal MRI ranges, and two CKD cohorts (n=24 each) to derive and validate a predictive MRI model against biopsy-confirmed fibrosis. The study uses an extensive 3T MRI protocol (T1, T2, T2*, diffusion, perfusion, and post-contrast mapping) and correlates imaging with blinded histopathology.

The study addresses an important unmet clinical need: non-invasive quantification of renal fibrosis. The rationale is strong, novelty lies in using post-contrast T1 mapping and a multiparametric composite marker, and the design includes an internal validation cohort. The protocol is scientifically sound, ethically approved, and well-structured.

Most issues relate to clarity, reporting completeness, and minor methodological precision rather than major flaws.

I have only minor concerns:

a) The manuscript mentions a “multiparametric MRI marker” but does not specify how it will be constructed. Pleas explicitly define whether this is a regression-based score, a weighted index, or another composite. Clarify how it will be generated in the derivation cohort and tested in the validation cohort (with prespecified performance criteria, e.g., R^2).

b) It is unclear how the 48 CKD patients are divided into derivation vs. validation cohorts; non-random allocation risks imbalance. Please state the allocation method and ensure groups are comparable (preferably randomized or stratified).

c) Eight predictors for only 24 derivation patients risks overfitting. Please describe methods to mitigate this (variable selection, penalized regression, collinearity checks) and commit to reporting adjusted R^2 with CIs.

d) Two MRI analysts are used, but handling of discrepancies and inter-rater reliability is not described. Please define whether results will be averaged, adjudicated, or consensus-based, and consider reporting ICCs.

e) The term “cortical contrast distribution measures” is vague.Please specify exactly what parameters will be extracted from post-contrast T1 mapping (e.g., ΔT1 at specific times, washout slope, AUC).

f) BMJ Open requires study dates; these are missing. Could the authors provide anticipated completion?

g) In terms of handling missing or poor-quality data, I noted that MRI failures or incomplete scans are possible but not addressed. Could the authors clarify handling rules (per-sequence exclusion vs. complete-case analysis, any imputation)?

h) Regarding confounding variables, we know that hydration, medications (diuretics, SGLT2i), and age may affect MRI values. Please clarify if these will be recorded and considered in sensitivity analyses or as covariates.

i) Regarding selection bias, only CKD patients who undergo biopsy are included. Note that this may limit generalizability to all CKD patients, since those not meeting biopsy criteria (e.g. more typical diabetic CKD cases) are not represented. The authors can argue that fibrosis is fibrosis, and the relationship with MRI should hold broadly, but it’s good to be transparent that the cohort is a selected one. Please add this as a limitation affecting generalizability.

j) In terms of pathology gold standard, two pathologists score fibrosis but no process is given for disagreement. It would be useful to state whether consensus, averaging, or a third reviewer will be used.

k) A 45-minute scan with multiple breath-holds may be challenging. Please describe how patient comfort will be managed (breaks, minimum acceptable sequences).

l) Minor issues such as “Masson trichome” (should be “trichrome”), inconsistent hyphenation in “T1 mapping,” and units formatting need correction. Please revise

terminology and formatting for consistency. Check that all acronyms (e.g., GBCA) are defined at first use.

VERSION 1 - AUTHOR RESPONSE

Reviewer Comment 1: This protocol describes the NIMBLE-CKD study, an observational cohort designed to validate multiparametric MRI biomarkers for assessing histology-proven renal fibrosis in chronic kidney disease (CKD). Three cohorts will be included: healthy volunteers (n=20) to define normal MRI ranges, and two CKD cohorts (n=24 each) to derive and validate a predictive MRI model against biopsy-confirmed fibrosis. The study uses an extensive 3T MRI protocol (T1, T2, T2*, diffusion, perfusion, and post-contrast mapping) and correlates imaging with blinded histopathology.

The study addresses an important unmet clinical need: non-invasive quantification of renal fibrosis. The rationale is strong, novelty lies in using post-contrast T1 mapping and a multiparametric composite marker, and the design includes an internal validation cohort. The protocol is scientifically sound, ethically approved, and well-structured.
Most issues relate to clarity, reporting completeness, and minor methodological precision rather than major flaws.

I have only minor concerns.

The manuscript mentions a “multiparametric MRI marker” but does not specify how it will be constructed. Pleas explicitly define whether this is a regression-based score, a weighted index, or another composite. Clarify how it will be generated in the derivation cohort and tested in the validation cohort (with prespecified performance criteria, e.g., R^2).

Response: We thank Reviewer 1 for their comments and valuable feedback particularly regarding the methodology of the study. This has now been clarified in the methods section of the manuscript, p. 10, line 31 – p. 11, line 11, as follows:

“For the primary outcome, a multivariable linear regression model including all MRI variables will be fitted to examine the association between the multiparametric MRI marker and

tubulointerstitial fibrosis. The model will be tested and developed in the CKD participant derivation cohort. Model performance will be assessed using a pre-specified minimum variance (R^2) threshold of 0.50 and $p<0.05$. […] A second independent CKD participant validation cohort will be used to evaluate the accuracy and sensitivity of the model using adjusted R^2."

Reviewer Comment 2: It is unclear how the 48 CKD patients are divided into derivation vs. validation cohorts; non-random allocation risks imbalance. Please state the allocation method and ensure groups are comparable (preferably randomized or stratified).

Response: This has now been clarified in the methods section of the manuscript, p. 6, lines 15-16, as follows:

"CKD participants will be recruited consecutively and randomly assigned into either the derivation or validation cohorts, respectively."

Furthermore, the baseline characteristics of each cohort will be reported and compared, to address any differences between groups. This has now been clarified in the methods, p. 7, line 7, as follows:

"Baseline characteristics for each cohort will be reported and compared."

Reviewer Comment 3: Eight predictors for only 24 derivation patients risks overfitting. Please describe methods to mitigate this (variable selection, penalized regression, collinearity checks) and commit to reporting adjusted R^2 with CIs.

Response: This has now been clarified in the methods section of the manuscript, p. 11, lines 11-14, as follows:

"The multivariable linear regression model will be derived using elastic net regularisation and nested re-sampling cross validation in order to minimise overfitting. The agreement performance of the model will be reported using adjusted R^2 with 95% confidence intervals."

Reviewer Comment 4: Two MRI analysts are used, but handling of discrepancies and inter-rater reliability is not described. Please define whether results will be averaged, adjudicated, or consensus-based, and consider reporting ICCs.

Response: This has now been clarified in the methods section of the manuscript, p. 9, lines 1-7, as follows:

"MR images will be analysed using the image analysis software Segment (Medviso, Lund, Sweden) by two independent observers blinded to the participant's baseline laboratory and biopsy results. The first analysis of observer 1 will be used for model derivation. Intra-observer variability will be evaluated and reported following second analysis by observer 1 of 5 healthy volunteers and 15 CKD participants (total n=20) that are randomly selected. Inter-observer variability will be evaluated in those same 20 participants comparing observer 1 with observer 2."

Reviewer Comment 5: The term "cortical contrast distribution measures" is vague. Please specify exactly what parameters will be extracted from post-contrast T1 mapping (e.g., ΔT1 at specific times, washout slope, AUC).

Response: : This has now been clarified in the methods section of the manuscript, p. 9, lines 17-23, as follows:

"Relative concentrations of the extracellularly distributing and renally excreted GBCA will be quantified in the renal cortex, renal medulla, and blood pool over time as ΔR1, where 1/T1 = R1 and ΔR1 = the difference between R1 before contrast and R1 at a given time point. These relative concentrations have not previously been quantified in health or disease in this manner, and the patterns of cortical contrast distribution dynamics will be evaluated in an exploratory manner using tissue kinetic modelling approaches for potential inclusion into the multivariable model."

Reviewer Comment 6: BMJ Open requires study dates; these are missing. Could the authors

provide anticipated completion?

Response: This has now been clarified in the study status section of the manuscript on p. 12, lines 1-2 as follows:

"Participant recruitment commenced in July 2024 and is anticipated to be completed by December 2028."

Reviewer Comment 7: In terms of handling missing or poor-quality data, I noted that MRI failures or incomplete scans are possible but not addressed. Could the authors clarify handling rules (per-sequence exclusion vs. complete-case analysis, any imputation)?

Response: This has now been clarified in the methods section of the manuscript on p. 11, lines 6-9 as follows:

"Patterns of missing data will be inspected and reported. Where appropriate, multiple imputation will be used to account for missing data under the missing at random assumption, and sensitivity analyses based on complete cases will be performed."

Reviewer Comment 8: Regarding confounding variables, we know that hydration, medications (diuretics, SGLT2i), and age may affect MRI values. Please clarify if these will be recorded and considered in sensitivity analyses or as covariates.

Response: We agree that these factors may confound MRI values, and have specified the collection and reporting of this data in the methods, p. 7, lines 1-6, as follows:

"Baseline characteristics of all participants including age, height, weight, serum creatinine and eGFR based on the CKD-EPI formula[32] will be collected and recorded. Additional clinical characteristics for CKD participants will be obtained from medical records including […] concomitant medications including diuretics, renin-angiotensin system inhibitors and sodium-glucose cotransporter-2 inhibitors."

We have also acknowledged this as a limitation in the discussion, p. 12, line 32 – p. 13, line 2, as follows:

"For example, experts have failed to reach consensus on factors such as the impact of medication and hydration or fasting status on MR images, and image analysis techniques."

In a study of this relatively limited size, it will be challenging to conduct meaningful statistical analyses adjusting for these variables. Other limitations include accurate assessment of hydration status and uncertainty around the effects of such medications including whether there is a dose-dependent effect. Thus, we will record and report these details, however additional sensitivity analyses are not planned.

Reviewer Comment 9: Regarding selection bias, only CKD patients who undergo biopsy are included. Note that this may limit generalizability to all CKD patients, since those not meeting biopsy criteria (e.g. more typical diabetic CKD cases) are not represented. The authors can argue that fibrosis is fibrosis, and the relationship with MRI should hold broadly, but it's good to be transparent that the cohort is a selected one. Please add this as a limitation affecting generalizability.

Response: This limitation has now been added to the discussion section of the manuscript on p. 13, lines 4-6, as follows:

"As the CKD participants will only include patients who have undergone kidney biopsy, there is potential risk for selection bias where patients with conditions that do not meet biopsy criteria are not represented, thus limiting generalisability."

Reviewer Comment 10: In terms of pathology gold standard, two pathologists score fibrosis but no process is given for disagreement. It would be useful to state whether consensus, averaging, or a third reviewer will be used.

Response: This has now been clarified in the methods section, p. 7, lines 15-21, as follows:

"All histopathological samples will be visually assessed and scored by two independent renal pathologists, who will be blinded to the MRI results. Tubulointerstitial fibrosis (TIF) will be assessed by Masson trichrome staining and quantified based on the percentage of cortex affected. The degree of tubulointerstitial fibrosis will be scored as a continuous variable from 0-100% and reported as the average of the two values from each pathologist. Based on this score, TIF will also be categorised based on the standard clinical scoring system (TIF 0 (<10%), TIF 1 (10-25%), TIF 2 (26-50%) or TIF 3 (>50%))."

Reviewer Comment 11: A 45-minute scan with multiple breath-holds may be challenging. Please describe how patient comfort will be managed (breaks, minimum acceptable sequences).

Response: To-date, 15 participants have been recruited to this study and completed the 45-minute kidney MRI. All participants have tolerated the MRI including the required breath-holds. Due to the difference sequences involved, there are adequate breaks in between each series of breath-holds.

Reviewer Comment 12: Minor issues such as "Masson trichome" (should be "trichrome"), inconsistent hyphenation in "T1 mapping," and units formatting need correction. Please revise terminology and formatting for consistency. Check that all acronyms (e.g., GBCA) are defined at first use.

Response: Spelling and formatting have now been corrected as follows:

- P. 7, line 17 *"Masson trichrome"*
- All hyphens have been removed for T1 and T2 mapping

All acronyms have been defined at first use

VERSION 2 - REVIEW

**Reviewer**	**1**
**Name**	**Torres, Ulysses**

Affiliation **Universidade Federal de São Paulo, Department of Radiology**

Date **09-Oct-2025**

COI

I have no further comments.
